# The implementation of physicians assistant in a surgical ward improves continuity in daily clinical work and increases comprehensibility of nurses and physicians

**DOI:** 10.1186/s13037-022-00344-7

**Published:** 2022-11-07

**Authors:** Sascha Halvachizadeh, Sarper Goezmen, Sibylle Schuster, Michel Teuben, Matthias Baechtold, Pascal Probst, Fabian Hauswirth, Markus K. Muller

**Affiliations:** 1 Department of Surgery, Spital Thurgau, Pfaffenholzstr. 4, 8501 Frauenfeld, Switzerland; 2 Department of medicine, University of Zurich, Rämistrasse 71, 8006 Zürich, Switzerland; 3 Cantonal Hospital Thurgau, Pfaffenholzstr. 4, 8501 Frauenfeld, Switzerland; 4 University of Zurich, Raemistrasse 71, 8006 Zürich, Switzerland

## Abstract

**Introduction:**

Physician Assistant (PA) have been deployed to increase the capacity of a team, supporting continuity and medical cover. The goal of this study was to assess the implementation of PAs on continuity of surgical rounds, on the collaboration of nurses and physicians and on support of administrative work.

**Methods:**

This cross-sectional survey was performed on nurses and physicians who work full-time at a surgical ward in a Swiss reference center. PAs were introduced in our institution in 2019. Participants answered a self-developed questionnaire 6 and 12 months after the implementation of PAs. Administrative work, teamwork, improvement of workflow, and training of physicians has been assessed. Participants answered questions on a 5-point Likert scale and were stratified according to profession (nurse, physician).

**Results:**

Participants (*n* = 53) reported a positive effect on the regular conduct of rounds (2.9, SD 1.1 points after 6 weeks and 3.5, SD 1.1 points after 12 weeks, *p* = 0.05). A significant improvement of nurse-doctor collaboration has been reported (3.6, SD 1.0 and 4.2, SD 0.8, p = 0.05). Nurses (*n* = 28, 52.8%) reported the that PAs are integrated in the physicians team rather than the nurses team (4.0, SD 0.0 points and 4.4, SD 0.7 points, *p* = 0.266) and a significant beneficial effect on the surgical clinic (3.7, SD 1.0 points and 4.4, SD 0.8 points, *p* = 0.043). Improved overall management of surgical cases was reported by the physicians (*n* = 25, 47.2%) (4.8, SD 0.4 and 4.3, SD 0.6, *p* = 0.046).

**Conclusion:**

The implementation of PA has improved the collaboration of physicians and nurses substantially. Continuity of rounds has improved and the administrative workload for residents decreased substantially. Overall, the implementation of PA was reported to be beneficial for the surgical clinic.

## Introduction

Physicians assistants (PAs), or Clinical nurse specialists (CNS) in Switzerland are a novel medical profession and are inspired by experiences from countries including the United States, Canada, or Australia [1–3].

The positive effect of PAs on routine clinical work has been reported in numerous studies [4]. The role of PAs in the ICU has been described as a valuable approach to improve interprofessionality [5]. There still is mixed evidence regarding the cost-effectiveness of PAs, however, the improvement of resource management following the implementation of PAs has been demonstrated before [6].

Although many critical issues such as the exact scope of practice or patient acceptance of PAs have been resolved, the PA profession remains young and continues to evolve [3]. Over time, patient acceptance has improved substantially, and the patient’s satisfaction of PAs is high [7]. PAs tend to see younger patients, usually less complex cases and have a different caseload than physicians. Furthermore PAs require supervision in selected cases. The training of PAs is constantly evolving and aims to bridge the gap between nurses and physicians [3, 8]. There still is a lack of evidence regarding the effect of the implementation of PAs on the core surgical team. Therefore, the goal of this study was to assess the implementation of PAs on continuity of surgical rounds in a European surgical reference center. Furthermore, the impact on the collaboration between nurses and physicians and the support in administrative work were analyzed.

## Methods

The reporting of this cross-sectional survey study follows the Consensus-Based Checklist for Reporting of Survey Studies (CROSS) [9]. A self-made questionnaire was distributed among nurses and physicians after 6 and 12 months following the implementation of PAs at a Swiss surgery reference institution.

### Ethical consideration

Nurses and physicians were fully informed about the survey and agreed to participate. With the return of the questionnaire, all participants approved further usage of anonymized data for (scientific) analyses and subsequent publication. An ethical approval was therefore waived.

### Data collection and questionnaire

The self-developed questionnaire consists of 18 questions and assesses the following points: continuity in daily surgical and administrative work (*n* = 8), teamwork and training (*n* = 10). Questions on daily surgical and administrative work included: subjective improvement of the amount of administrative work, continuity in ward rounds, improved discharge management, relief in routine “ward-workload”; The believe whether PAs are a decent addition to the surgical clinic or not; and if implementation of PAs result in improved efficiency during rounds.

Teamwork and training included the assessment of: more time in the operating theater, more time in outpatient clinic, improvement of the collaboration of nurses and physicians, improvement of weekend-shift; requirement of more training, integration in team (nurse and physicians), interdisciplinary acceptance, improvement of training.

### Survey administration

The PAs were implemented in 2019 and 6 (early survey) and 12 (late survey) months after the implementation nurses, and physicians working at the surgical ward, were asked to fill out the self-administered questionnaire. The data were manually transferred to a digital working sheet. To minimize human error in data entry, each data point was controlled by a second transcriber. A prior training or study preparation was not warranted.

### Surgical reference center and sample characteristics

The Swiss surgical reference institution employs 14 to 15 residents per year. The center employed 2 PAs, both with a nursing background.

All included participants worked at least 3 years full-time equivalent at the surgical ward. The PAs are enrolled in a structured Certificate of Advanced Studies (CAS) training program.

### Statistical analyses

Continuous variables are presented as mean and standard deviation (SD), categorical variables as count and percentage. Group comparisons (6 weeks and 12 weeks) was performed with paired t-test on continuous variables and chi-squared test for categorical variables. Data of nurses and physicians are presented separately. The Likert scale was transformed into a continuous scale to improve comparability and readability. All statistical analyses were performed with R [10].

## Results

### Overall effect of the implementation of PAs

**Fig. 1:**
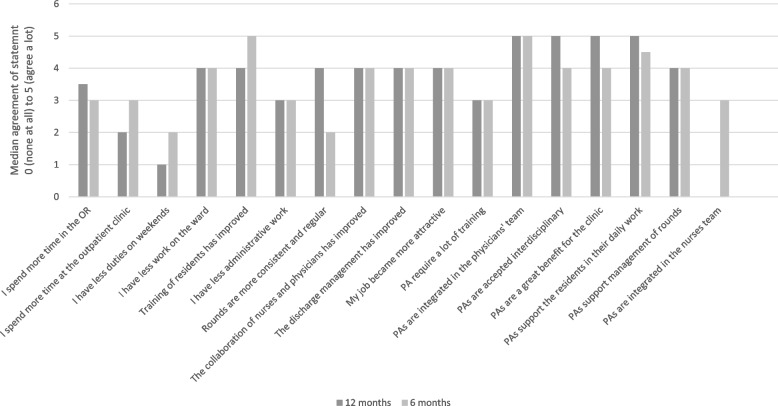
The medical personell answered questions 6 and 12 months after the implementation of Physician Assistants (PAs) on a 5-point Likert scale

The participants (*n* = 52) reported an improvement of the organization of ward (4.0, SD 0.9points after 6 months, and 4.2, SD 0.8points, after 12 months, *p* = 0.418). Further, the consistency of rounds has improved over 6 months (2.8, SD 1.1points and 3.5. SD 1.1points, *p* = 0.05). A high interdisciplinary acceptance of the PAs was reported (4.2, SD 0.9 points and 4.4, SD 0.7 points, *p* = 0.363) as well as a great team integration (4.7, SD 0.5 points, and 4.7, SD 0.6 points, *p* = 0.942). The collaboration of nurses and physicians improved substantially after the implementation of PAs (3.6, SD 1.0 points and 4.2, SD 0.8 points, p = 0.05). The administrative work, and the work at the ward were more or less affected by PAs (3.0, SD 1.2 points and 3.1, SD 1.3 points, p 0 0.841). The highest improvement following PAs implementation were reported to be support of physicians (4.6, SD 0.5 points) (Fig. 1).

### Effect on the nursing staff

**Fig. 2:**
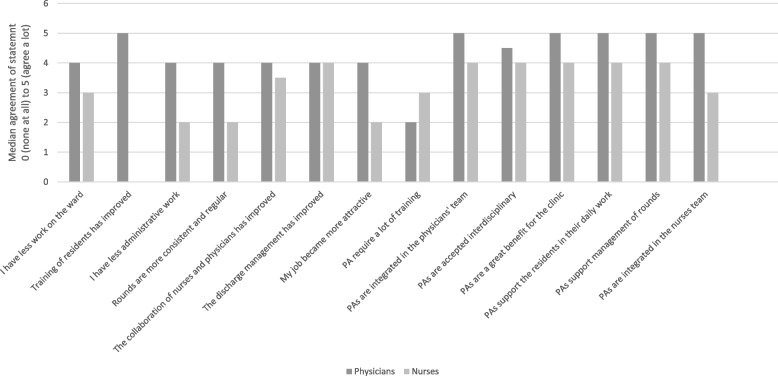
A comparison of answers of physicians and nurses 6 months after the implementation of PAs

Nurses (*n* = 28, 52.8%) reported PAs to be a part of the physicians team rather than the nurses team (4.0, SD 0.0 points and 4.4, SD 0.7 points, *p* = 0.266). The statistical significant beneficial effect of the implementation of PAs on the surgical clinic was further reported by the nursing staff (3.7, SD 1.0 points and 4.4, SD 0.8 points, *p* = 0.043). The implementation of PAs had did not affect the administrative work of the nurses (2.4, SD 0.7 points and 2.7, SD 1.2, *p* = 0.441), however, the nurses reported a significantly beneficial support of PAs for the residents (4.0, SD 0.8 points and 4.6, SD 0.7, *p* = 0.05) (Fig. 2).

### Effect on medical staff

**Fig. 3:**
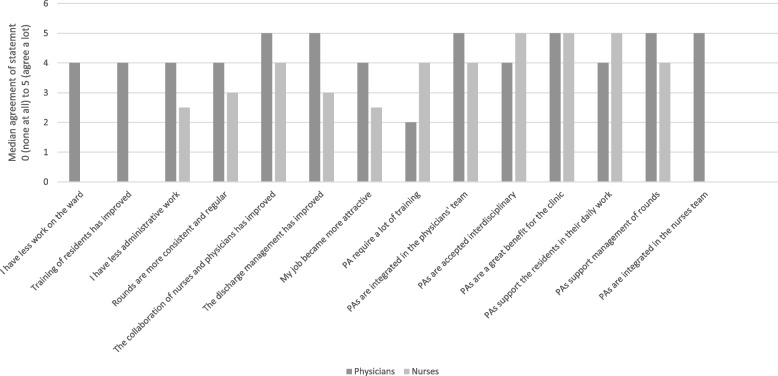
A comparison of answers of physicians and nurses 12 months after the implementation of PAs

Participating physicians (*n* = 25, 47.2%) reported the PAs to be a great support during regular rounds (4.7, SD 0.5 and 4.5, SD 0.8, *p* = 0.332). Physicians further reported Pas to be a great support for the residents (4.8, SD 0.4 and 4.3, SD 0.65 *p* = 0.046). The interdisciplinary acceptance (4.4, SD 0.7 and 4.3, SD 0.7, *p* = 0.719) and the team integration to the medical team were very high (4.9. SD 0.3 and 4.9, SD 0.3, *p* = 0.907). Over time the collaboration of nurses and physicians improved significantly following the implementation of PAs (4.0, SD 1.1 points and 4.6, SD 0.5 points, *p* = 0.049). Further, the implementation of PAs improved discharge management (4.3, SD 0,7 points, and 4.4, SD 0.8 points, *p* = 0.693) (Fig. 3).

There were no significant different reports comparing attending and residents.

## Discussion

The implementation of PAs in Swiss surgical clinics is a novel approach that aims to improve interdisciplinary work and routine workflow. The present study aimed to assess the effect of implementation of PAs in a European reference center on nurses and physicians after 6 and 12 months following the implementation. The main results included:

- The implementation of PAs improved the collaboration of nurses and physicians substantially
- PAs improve the continuity and the organization of routine surgical rounds, as well as discharge management
- The positive effect of routine PA-involvement on clinical wards was reported by both nurses and physicians

Task-shifting of physicians work from physicians to nurses aimed to tackle growing doctor shortage and reduce physicians workload, as well as human resource costs [11]. Task-shifting is discussed rigorously in the literature, however, evidence supporting task-shifting is scarce [12]. Nevertheless, PAs have been implemented to fill the gap between nurses and physicians. The interprofessional collaboration of nurses and physicians is a huge issue that healthcare organizations invest a lot of resources on [13]. It has been shown that nurses and physicians view collaboration in a different way, and this may impair the quality of this collaboration [14]. PAs usually have a nursing background, might however have any other medical background (e.g. physiotherapist, speech therapists, ergo-therapists, and so on). A second prerequisite for PAs is the completion of a higher education to gain more profound medical knowledge [15, 16]. With this training, PAs bridge the gap between nurses and physicians and might therefore improve collaboration. In contrast to other countries, the PAs are integrated in the physician’s team, and share the same office space. Following the present results, bothe the nurses and the physicians agree on the role of PAs. Further, both PAs have a nursing background. The PAs completed their primary training in nursing school and worked as nurses for several years. Afterwards they proceeded to become PAs and supported the physician’s team. The background might further be one potential reason that supports collaboration of nurses and physicians.

The role of PAs is expanding globally. Despite only working during daytime hours on weekdays, PAs are involved in more than half of all patient encounters, and lead most of the multidisciplinary meetings [1]. The integration of PAs provides consistency and improves efficiency of management of war issues [1]. PAs focus mostly on routine cases and provide more time for the residents to investigate complicated cases in more detail. This leads to improved, standardized discharge management, planning, timely consultation and effective organization [1]. The current study shows these assumed effects in a cross-sectional study on multiple involved medical professions at a surgical department at an European reference hospital.

Overall, this study suggests that the implementation of PAs has a positive effect on the surgical clinical. This includes the aforementioned improved collaboration, and the improved management of daily ward work. PAs have been shown to indirectly benefit the medical health care system by optimizing patient management [17]. The comparability of results conducted after 6 and 12 months indicate that PAs are accepted early and persistently as a support for the medical staff.

### Limitations

One limitation of the present study is the lack of the use of standardized questionnaires. However, the goal was to assess the effect of the implementation of PAs at our Swiss surgical reference center, and therefore the questionnaire was designed specifically for the local issues at hand. As no validated questionnaires have been developed before, a novel questionnaire was developed. Second, one might argue that local training of PAs is required prior to analyzing the effect. We therefore performed the survey 6 and 12 months after the implementation and found comparable results, suggesting that the PAs are integrated very early into the medical team.

## Conclusion

The present data show the beneficial effect of the introduction of PAs on the surgical team. This beneficial effect becomes more profound over time and includes improved nurse-doctor collaboration, improved patient management, consistency of medical rounds and better discharge management. In a time of ongoing standardization of health care, PAs will play an increasing role in surgical care. Both subjective analysis and objective studies on the impact of this trend are mandated to optimize the benefit of this development.

## Data Availability

The data is available on reasonable request.

## References

[1] Lack A, Saddik M, Engels P, Lethbridge S, Nenshi R (2020). The emergence of the physician assistant role in a Canadian acute care surgery setting. Can J Surg.

[2] Hooker RS, Hogan K, Leeker E (2007). The globalization of the physician assistant profession. J Physician Assist Educ.

[3] Larson EH, Hart LG (2007). Growth and change in the physician assistant workforce in the United States, 1967-2000. J Allied Health.

[4] Halter M, Drennan V, Chattopadhyay K, Carneiro W, Yiallouros J, de Lusignan S (2013). The contribution of physician assistants in primary care: a systematic review. BMC Health Serv Res.

[5] Gabbard ER, Klein D, Vollman K, Chamblee TB, Soltis LM, Zellinger M (2021). Clinical nurse specialist: a critical member of the ICU team. Crit Care Med.

[6] Salamanca-Balen N, Seymour J, Caswell G, Whynes D, Tod A (2018). The costs, resource use and cost-effectiveness of clinical nurse specialist-led interventions for patients with palliative care needs: a systematic review of international evidence. Palliat Med.

[7] Hooker RS, Moloney-Johns AJ, McFarland MM (2019). Patient satisfaction with physician assistant/associate care: an international scoping review. Hum Resour Health.

[8] Tsyrulnik A, Goldflam K, Coughlin R, Wong AH, Ray JM, Bod J (2020). Implementation of a physician assistant emergency medicine residency within a physician residency. West J Emerg Med.

[9] Sharma A, Minh Duc NT, Luu Lam Thang T, Nam NH, Ng SJ, Abbas KS (2021). A consensus-based checklist for reporting of survey studies (CROSS). J Gen Intern Med.

[10] Team RC, Others. R: A language and environment for statistical computing 2013; Available from: http://r.meteo.uni.wroc.pl/web/packages/dplR/vignettes/intro-dplR.pdf.

[11] Chopra M, Munro S, Lavis JN, Vist G, Bennett S (2008). Effects of policy options for human resources for health: an analysis of systematic reviews. Lancet..

[12] Karimi-Shahanjarini A, Shakibazadeh E, Rashidian A, Hajimiri K, Glenton C, Noyes J (2019). Barriers and facilitators to the implementation of doctor-nurse substitution strategies in primary care: a qualitative evidence synthesis. Cochrane Database Syst Rev.

[13] Siedlecki SL, Hixson ED (2015). Relationships between nurses and physicians matter. Online J Issues Nurs.

[14] Sollami A, Caricati L, Sarli L (2015). Nurse-physician collaboration: a meta-analytical investigation of survey scores. J Interprof Care.

[15] Wu F (2019). Emergency medicine physician assistant (EMPA) postgraduate training programs: program characteristics and training curricula. West J Emerg Med.

[16] Hix LR, Fernandes SM (2020). An initial exploration of the physician assistant role in Germany. J Physician Assist Educ.

[17] Althausen PL, Shannon S, Owens B, Coll D, Cvitash M, Lu M (2013). Impact of hospital-employed physician assistants on a level II community-based orthopaedic trauma system. J Orthop Trauma.

